# Supplementary material for: Modeling glioblastoma heterogeneity as a dynamic network of cell states
Source: Mol Syst Biol. 2021 Sep 16;17(9):e10105. doi: 10.15252/msb.202010105 (PMC8444284; doi:10.15252/msb.202010105)
Supplement: Supplementary file 6 — Source Data for Figure 5 [file MSB-17-e10105-s004.zip › Figure5A_sourcedata/GSEA_3017/nosigdb_stateA.GseaPreranked.1622795026485/OLSEN_SCPLIKE.html]

Details for gene set OLSEN\_SCPLIKE[GSEA]

|  || Dataset | state53017 |
| Phenotype | NoPhenotypeAvailable |
| Upregulated in class | na\_neg |
| GeneSet | OLSEN\_SCPLIKE |
| Enrichment Score (ES) | -0.57801884 |
| Normalized Enrichment Score (NES) | -2.7513657 |
| Nominal p-value | 0.0 |
| FDR q-value | 0.0 |
| FWER p-Value | 0.0 |
Table: GSEA Results Summary

  

Fig 1: Enrichment plot: OLSEN\_SCPLIKE      
 Profile of the Running ES Score & Positions of GeneSet Members on the Rank Ordered List

  

| PROBE | GENE SYMBOL | GENE\_TITLE | RANK IN GENE LIST | RANK METRIC SCORE | RUNNING ES | CORE ENRICHMENT || 1 | VIM |  |  | 46 | 0.543 | -0.0218 | No |
| 2 | S100A16 |  |  | 156 | 0.404 | -0.1166 | No |
| 3 | ITGB1 |  |  | 256 | 0.346 | -0.2037 | No |
| 4 | PLP1 |  |  | 272 | 0.338 | -0.2030 | No |
| 5 | MT2A |  |  | 473 | 0.275 | -0.3997 | No |
| 6 | ZFP36L1 |  |  | 532 | 0.264 | -0.4478 | No |
| 7 | ITGA6 |  |  | 544 | 0.261 | -0.4466 | No |
| 8 | CD99 |  |  | 604 | -0.252 | -0.4963 | No |
| 9 | VMP1 |  |  | 632 | -0.262 | -0.5118 | No |
| 10 | S100B |  |  | 696 | -0.299 | -0.5635 | Yes |
| 11 | MALAT1 |  |  | 702 | -0.302 | -0.5540 | Yes |
| 12 | S100A6 |  |  | 717 | -0.313 | -0.5534 | Yes |
| 13 | ZEB2 |  |  | 722 | -0.319 | -0.5421 | Yes |
| 14 | SPTBN1 |  |  | 725 | -0.322 | -0.5285 | Yes |
| 15 | NTM |  |  | 762 | -0.354 | -0.5490 | Yes |
| 16 | ITGB8 |  |  | 774 | -0.363 | -0.5429 | Yes |
| 17 | IGFBP7 |  |  | 780 | -0.368 | -0.5302 | Yes |
| 18 | SPARC |  |  | 782 | -0.369 | -0.5133 | Yes |
| 19 | PNRC1 |  |  | 786 | -0.371 | -0.4984 | Yes |
| 20 | APP |  |  | 812 | -0.394 | -0.5054 | Yes |
| 21 | DST |  |  | 818 | -0.400 | -0.4912 | Yes |
| 22 | CD59 |  |  | 831 | -0.422 | -0.4832 | Yes |
| 23 | IFITM2 |  |  | 864 | -0.460 | -0.4944 | Yes |
| 24 | ANXA2 |  |  | 872 | -0.482 | -0.4783 | Yes |
| 25 | GAS7 |  |  | 875 | -0.490 | -0.4565 | Yes |
| 26 | PMEPA1 |  |  | 877 | -0.497 | -0.4334 | Yes |
| 27 | GPM6B |  |  | 897 | -0.542 | -0.4269 | Yes |
| 28 | CST3 |  |  | 933 | -0.680 | -0.4305 | Yes |
| 29 | IFITM3 |  |  | 954 | -0.830 | -0.4111 | Yes |
| 30 | CNN3 |  |  | 961 | -0.883 | -0.3743 | Yes |
| 31 | CRYAB |  |  | 967 | -0.940 | -0.3337 | Yes |
| 32 | LGALS3 |  |  | 970 | -0.989 | -0.2876 | Yes |
| 33 | ANXA1 |  |  | 972 | -1.018 | -0.2391 | Yes |
| 34 | PMP22 |  |  | 982 | -1.519 | -0.1745 | Yes |
| 35 | IGFBP5 |  |  | 983 | -1.595 | -0.0967 | Yes |
| 36 | NEAT1 |  |  | 986 | -2.049 | 0.0011 | Yes |
Table: GSEA details [plain text format]

  

Fig 2: OLSEN\_SCPLIKE: Random ES distribution      
 Gene set null distribution of ES for **OLSEN\_SCPLIKE**
